# In Vitro Degradation and Photoactivated Antibacterial Activity of a Hemin-CaP Microsphere-Loaded Coating on Pure Magnesium

**DOI:** 10.3390/jfb14010015

**Published:** 2022-12-26

**Authors:** Lixin Long, Yang Song, Xiaoyi Tian, Lanyue Cui, Chengbao Liu, Shuoqi Li, Yu Wang, Rongchang Zeng

**Affiliations:** 1 College of Materials Science and Engineering, Shandong University of Science and Technology, Qingdao 266590, China; 2 College of Pharmaceutical Sciences, Soochow University, Suzhou 215123, China; 3 School of Materials Science and Engineering, Zhengzhou University, Zhengzhou 450002, China

**Keywords:** magnesium, degradable biomaterial, antibacterial coating, biocompatibility, hemin

## Abstract

Photoactivated sterilization has received more attention in dealing with implant-associated infections due to its advantages of rapid and effective bacteriostasis and broad-spectrum antibacterial activity. Herein, a micro-arc oxidation (MAO)/polymethyltrimethoxysilane (PMTMS)@hemin-induced calcium-bearing phosphate microsphere (Hemin-CaP) coating was prepared on pure magnesium (Mg) via MAO processing and dipping treatments. The morphology and composition of the coating were characterized via scanning electron microscopy, Fourier transform infrared spectrometer, X-ray diffractometer and X-ray photoelectron spectrometer. Corrosion behavior was evaluated through electrochemical and hydrogen evolution tests. The release of Fe^3+^ ions at different immersion times was measured with an atomic absorption spectrophotometer. Antibacterial performance and cytotoxicity were assessed using the spread plate method, MTT assay and live/dead staining experiment. The results showed that the corrosion current density of the MAO/PMTMS@(Hemin-CaP) coating (4.41 × 10^−8^ A·cm^−2^) was decreased by two orders of magnitude compared to that of pure Mg (3.12 × 10^−6^ A·cm^−2^). Photoactivated antibacterial efficiencies of the Hemin-CaP microspheres and MAO/PMTMS@(Hemin-CaP) coating reached about 99% and 92%, respectively, which we attributed to the photothermal and photodynamic properties of hemin with a porphyrin ring. Moreover, based on the release of Fe^3+^ ions, the MC3T3-E1 pre-osteoblasts’ viability reached up to 125% after a 72 h culture, indicating a positive effect of the coating in promoting cell growth. Thus, this novel composite coating holds a promising application as bone implants.

## 1. Introduction

Magnesium (Mg)-based alloys have attracted great interest in the temporary orthopaedic implant field owing to their remarkable biodegradability, excellent biocompatibility and similar elastic modulus and density with the natural bone [1,2,3]. Furthermore, Mg^2+^ ions produced during the degradation of Mg-based alloys could stimulate new bone growth [4]. The unfortunate complication is that Mg-based alloys can degrade too rapidly under physiological conditions, losing mechanical strength before the tissue has fully healed and producing hydrogen in the degradation process at a fast rate that the host tissue may be unable to deal with [5]. On the other hand, although the increased pH value induced by in vitro degradation of Mg-based alloys has exhibited antibacterial activity, the powerful buffering capacity of our human body would weaken this antibacterial effect [6,7], which is not enough to handle the complex implant-associated infections (IAIs). Thus, it is vital to prepare functional coatings on Mg-based alloys to enhance both their corrosion resistance and their antibacterial activity.

Among various preparation methods of coatings such as micro-arc oxidation (MAO) [8,9], electrophoretic deposition [10,11], layer-by-layer assembly [12,13], chemical vapor deposition [14] and chemical conversion [15,16], MAO has been extensively used for surface modification of Mg-based alloys because of its remarkable wear resistance as well as high hardness [17]. However, the MAO coating with porous structures is vulnerable to penetration by a corrosive medium. Hence, it is necessary to adopt measures to seal the porous structures, one of which is the commonly used fabrication of polymer coatings after the MAO process [18]. Jia et al. [19] prepared chitosan multilayers loaded with cerium trivalent on the MAO surface of a Mg-1Ca alloy using spin-coating. This biocompatible ceramic/polyelectrolyte anticorrosion system possessed a pH-responsive self-healing property. Lu et al. [20] prepared a poly-L-lactic acid (PLLA) coating on an MAO-pretreated AZ81 alloy and then fabricated a drug-release coating consisting of a poly (DL-lactide-co-glycolide)/paclitaxel (PLGA/PTX) layer and a pure PLGA layer. The results revealed that the PLLA efficiently sealed the micropores and microcracks of the MAO coating and a nearly linear sustained-release of PTX was achieved without significant burst release. Ghanbari et al. [21] deposited a poly trimethylene carbonate polymer layer to seal open pores of the MAO layer and functionalized it with a biomimetic polydopamine layer. The multilayer coating promoted the formation of hydroxyapatite and cell adhesion on the surface. Our previous work [22] showed that polymethyltrimethoxysilane (PMTMS) can also be used as a polymer coating due to its excellent film formability and durability. The hydrolyzed silane condensed with the hydroxyl groups of the MAO coating sealed its porous structures effectively and enhanced its corrosion resistance. In addition, loading drugs [23] or nanoparticles [24] with the PMTMS has also been proved to be feasible.

As one of the prime reasons for orthopedic surgery failure, IAI has become an arduous challenge. IAIs can be induced by various contamination sources, including inadequate disinfection of surgical instruments, inadequate cleaning of the operating room environment, hypothermia of patients during anesthesia and contamination from patient’s skin and mucous membranes [25]. At present, antibiotics are the most-used agents to treat IAIs. Nevertheless, the overuse of antibiotics will give rise to increasingly severe bacterial resistance [26,27]. Therefore, it is urgent to seek new non-antibiotic agents and strategies that will kill bacteria without generating bacterial resistance. Photoactivated sterilization has received more attention in recent years due to its advantages of rapid and effective bacteriostasis and broad-spectrum antibacterial activity [28]. To date, a variety of photo-responsive materials, such as inorganic nanoparticles [29,30], layered double hydroxides [31,32], cyanine-based agents [33,34], porphyrin-based agents [35] and diketone-based agents [36] have been attempted to fabricate antibacterial coatings on the surface of bone implants to deal with IAIs. Thereinto, porphyrins and porphyrin derivatives have considerable potential in biomedical applications because of their distinctive light-harvesting properties and photophysical properties [37]. For instance, their porphyrin macrocycles can transform near-infrared (NIR) light into heat for photothermal therapy (PTT) and produce reactive oxygen species (ROS) for photodynamic therapy (PDT) [35,38]. Our prior research [35] prepared a sodium copper chlorophyllin induced calcium-bearing phosphate coating on the Mg alloy AZ31 through layer-by-layer assembly. The antibacterial efficacy of the prepared coating against *S. aureus* and *E. coli* reached 99.8% and 99.9% in vitro through the combination of PTT, PDT and Cu^2+^ ion release. However, the release of Cu^2+^ ions during coating degradation, especially the burst release within 24 h, would lead to galvanic corrosion and accelerate the failure of the coating.

Compared with the standard electrode potential of Cu (*E*(Cu^2+^/Cu) = 0.3419 V), the electrode potential difference between Fe (*E*(Fe^3+^/Fe) = −0.037 V) and Mg (*E*(Mg^2+^/Mg) = −2.372 V) is smaller [39]. Therefore, the tendency of galvanic corrosion between Fe-containing materials and Mg-based implants is lower than that of Cu-containing materials. Fe, as the highest trace metal element with about 4 g in the human body, plays vital roles in vivo, including the storage, transport and activation of molecular oxygen, the decomposition of peroxides, the reduction of ribonucleotides and dinitrogen and the transport of electrons [40]. As an Fe-containing porphyrin (hemoglobin protein) extracted from the red blood cells, hemin has been recognized as an ideal iron supplement with proven biosafety [41,42]. In recent years, hemin has also been widely utilized for biosensors and cancer therapy owing to the electron-rich π-system of the cyclic tetrapyrrole and the catalytic function of the chelated ferric iron [43,44]. For example, Dang et al. [42] fabricated multifunctional scaffolds via loading hemin and doxorubicin on bioactive glass ceramics, which could inhibit tumor growth in vivo through the combination of PTT and chemotherapy. However, few researchers have reported using hemin coating on the surface of Mg-based implants for anti-infection treatment through PTT and PDT up to now.

Calcium-bearing phosphates (CaP), including monocalcium phosphate (MCP, Ca(H_2_PO_4_)_2_), dicalcium phosphate (DCP, CaHPO_4_), tricalcium phosphate (TCP, Ca_3_(PO_4_)_2_), oxyapatite (OA, Ca_10_(PO_4_)_6_O), hydroxyapatite (HA, Ca_10_(PO_4_)_6_(OH)_2_), octacalcium phosphate (OCP, Ca_8_(HPO_4_)_2_(PO_4_)_4_·5H_2_O) and tetracalcium phosphate (TTCP, Ca_4_(PO_4_)_2_O), are chemically analogous to the mineral composition of bones and have been generally used as Mg-based alloy coatings owing to their superior osseointegration and biocompatibility [45,46]. Previous studies have shown that organic molecules and polymers containing carboxyl groups, such as EDTA [47], amino acids [48] and polyacrylic acid [12] can induce the deposition of CaP through molecular recognition [2]. Thus, hemin may also be a good candidate to induce CaP deposition via molecular recognition with carboxyl groups.

In this study, a PMTMS@hemin-induced CaP coating was prepared on MAO-coated pure Mg via dipping treatments, in which hemin acted as both photosensitizer and inducer of molecular recognition, to achieve good photoactivated antibacterial properties, long-term corrosion resistance and excellent biocompatibility. Above all, the mechanisms of coating formation, degradation and antibacterial activity were discussed.

## 2. Materials and Methods

### 2.1. Materials

Extruded plates of pure Mg (99.97% purity), provided by Shandong Yinguang Yuyuan Light Metal Precise Forming Co., Ltd., Shandong, China, were used as substrates and cut into cuboids with dimensions of 20 mm × 20 mm × 4 mm (used for corrosion characterization) and 10 mm × 10 mm × 4 mm (used for antibacterial and cytotoxicity tests) with a linear cutting machine. The chemical reagents used in this study are as follows: hemin (95%, Shanghai Aladdin Biochemical Technology Co., Ltd., Shanghai, China), methyltrimethoxysilane (MTMS, 98%, Shanghai Macklin Biochemical Co., Ltd., Shanghai, China), N, N-dimethylformamide (DMF, AR, Sinopharm Chemical Reagent Co., Ltd., Shanghai, China), CaCl_2_ (AR, Tianjin Guangfu Technology Development Co., Ltd., Tianjin, China), NaH_2_PO_4_ (AR, Xiya Chemical Technology Co., Ltd., Chengdu, China), NaOH (AR, Chengdu Kelong Chemical Co., Ltd., Chengdu, China), Na_2_SiO_3_·9H_2_O (AR, Tianjin Beichen District Fangzheng Reagent Factory, Tianjin, China) and Na_2_B_4_O_7_·10H_2_O (AR, Tianjin Guangfu Technology Development Co., Ltd., Tianjin, China).

### 2.2. Preparation of Hemin-CaP Microspheres

Hemin-CaP microspheres were prepared via a solvothermal method. Samples of 2 mM CaCl_2_, 1.2 mM NaH_2_PO_4_ and 0.4 mM hemin were added into 30 mL DMF under vigorous stirring. The solution was then moved into a stainless-steel autoclave with a Teflon liner and reacted at 140 °C for 12 h. The obtained microspheres were separated from the solution by centrifugation, washed three times with DI water and dried at 80 °C, then ground by an agate mortar. Finally, a brown Hemin-CaP powder was obtained.

### 2.3. Preparation of MAO Coatings

The substrates were progressively ground with sandpaper to 1500 grit, followed by cleaning with DI water and ethanol and drying with warm air. Subsequently, MAO coatings were formed using a home-built setup that included an HNMAO-20A-DPM400 power supply, a motor agitator and an acrylic box containing a stainless-steel plate (cathode). The parameters of the MAO process were as follows: frequency 200 Hz, duty ratio 10%, voltage 300 V and oxidation time 5 min. The electrolyte contained 5 g/L of NaOH, 7 g/L of Na_2_SiO_3_·9H_2_O and 9 g/L of Na_2_B_4_O_7_·10H_2_O. The whole MAO process was carried out at room temperature and the voltage was increased gradually from 180 V to 300 V. The breakdown voltage and breakdown time were about 280 V and 1 min, respectively. Afterward, the samples were cleaned with DI water and dried in an oven.

### 2.4. Preparation of MAO/PMTMS@(Hemin-CaP) Coatings

**Figure 1:**
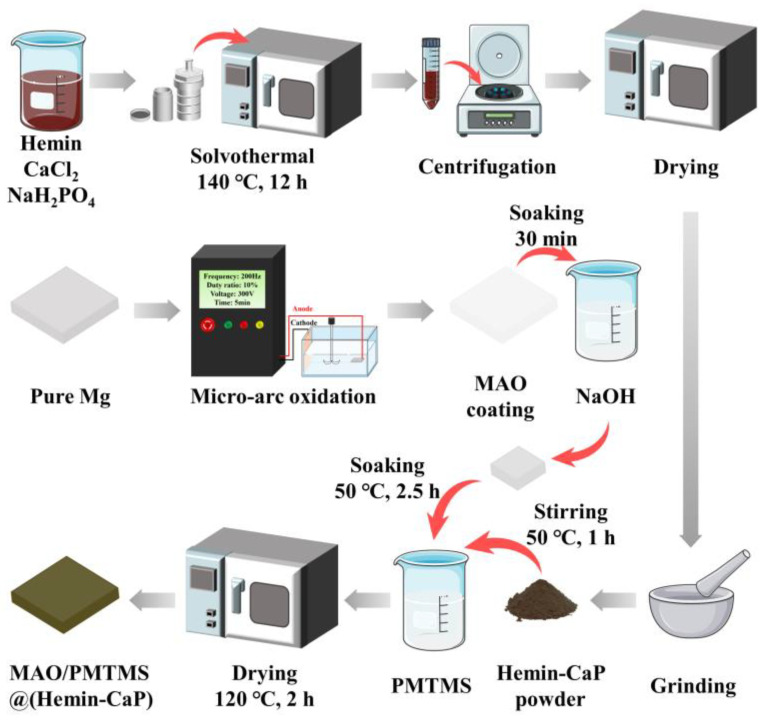
Flowsheet for the preparation of MAO/PMTMS@(Hemin-CaP) coating on pure Mg.

The MAO coatings were soaked in 1 M NaOH solution for 30 min to acquire the hydroxylated MAO coatings. A solution of MTMS doped with Hemin-CaP microspheres was prepared by adding 60 mg Hemin-CaP powder into MTMS solution (9 mL MTMS, 30 mL ethanol, and 60 mL DI water), followed by stirring at 50 °C for 1 h. The hydroxylated MAO coatings were soaked in the MTMS solution doped with Hemin-CaP at 50 °C for 2.5 h. Finally, the MAO/PMTMS@(Hemin-CaP) coatings were acquired after cleaning with DI water and drying in an oven at 120 °C for 2 h. The preparation procedure is illustrated in Figure 1.

### 2.5. Surface Analysis

The morphology and micro-structure of the powder were observed via scanning electron microscopy (SEM, Apreo S HiVac, Thermo Fisher Scientific, USA) and transmission electron microscopy (TEM, Talos F200X G2, Thermo Fisher Scientific, USA). The surface and cross-sectional morphologies of the coatings were observed via scanning electron microscopy (SEM, Nova NanoSEM 450, FEI, USA). The elemental composition was investigated using energy dispersive X-ray spectroscopy (EDS) which was connected to the TEM and SEM. The chemical bonding states were characterized via Fourier transform infrared spectrometer (FT-IR, Nicolet 380, Thermo Fisher Scientific, USA) and X-ray photoelectron spectrometer (XPS, Nexsa, Thermo Fisher Scientific, USA). The crystallographic structures were analyzed with an X-ray diffractometer (XRD, D/Max 2500PC, Rigaku, Japan). The water contact angle was measured via a contact angle measuring instrument (JC2000D1, Shanghai Zhongchen Digtal Technology Apparatus Co., Ltd., China).

### 2.6. Corrosion Characterization

Electrochemical impedance spectroscopy (EIS) and potentiodynamic polarization curves were evaluated with an electrochemical workstation (PARSTAT2273, AMETEK, USA). A typical three-electrode system consisted of a saturated calomel electrode as reference electrode, a platinum sheet as counter electrode, and a sample with an exposure area of 1 cm^2^ as working electrode applied in Hank’s solution (8.0 g/L NaCl, 0.4 g/L KCl, 0.14 g/L CaCl_2_, 0.35 g/L NaHCO_3_, 1.0 g/L glucose, 0.1 g/L MgCl_2_·6H_2_O, 0.06 g/L MgSO_4_·7H_2_O, 0.06 g/L KH_2_PO_4_ and 0.06 g/L Na_2_HPO_4_·12H_2_O) at ambient temperature. The open circuit potentials were performed for 10 min before the EIS measurements. The interference potential of EIS and the scan rate of potentiodynamic polarization curves were 10 mV and 2 mV/s, respectively. ZSimpWin and PowerSuite were used to fit the equivalent circuits (EC) of EIS and the electrochemical parameters (i.e., corrosion potential (*E*_corr_), corrosion current density (*i*_corr_), anode Tafel slope (*β*_a_) and cathode Tafel slope (*β*_c_)) of the potentiodynamic polarization curves, respectively. Polarization resistance (*R*_p_) was calculated according to the Stern-Geary equation [49]:

(1) $$R_{p}=\frac{\beta_{a}{\;\times \;\beta }_{c}}{2{.303\;\times \;i}_{\mathrm{corr}}\;\times \;\left(\beta_{a}+\beta_{c}\right)}$$

Additionally, the hydrogen evolution test was measured by putting the samples into Hank’s solution at 37 °C under an upside-down funnel linked to an inverted buret and recording the scale of the buret periodically for 430 h. To investigate the degradation process of the MAO/PMTMS@(Hemin-CaP) coating, the morphology and composition of the coating were measured after immersions for 75, 150, 225 and 430 h. The Fe element released from the coatings was detected with anf atomic absorption spectrophotometer (TAS-986F, PERSEE, China). All samples were tested in triplicate.

### 2.7. Photothermal and Photodynamic Tests

The photothermal performance of the samples was detected in air and Hank’s solution (700 μL) using an 808 nm NIR laser beam (P1, Hi-Tech Optoelectronics Co., Ltd., China) with a power density of 1.6 W/cm^2^. The temperatures were captured using an infrared thermal imager (PTi120, Fluke). In order to calculate the photothermal conversion efficiency (*η*) of the MAO/PMTMS@(Hemin-CaP) coating, its absorbance at 808 nm was measured with an ultraviolet-visible/near-infrared spectrophotometer (UV-Vis/NIR, UH4150, Hitachi, Japan), and the specific calculation steps are described in the supporting information. For the photodynamic test, the electron spin resonance spectrometer (ESR, EMXplus, BRUKER, Germany) was employed to detect the ROS (^1^O_2_) produced by samples using 2, 2, 6, 6-tetramethylpiperidine (TEMP) as a capture agent. For the Hemin-CaP powder, 100 μL suspension (500 μg/mL) was mixed with 5 μL TEMP solution (100 mM) and then exposure to 808 nm NIR light for 10 min. For the MAO/PMTMS@(Hemin-CaP) coating, the sample was immersed in DI water for 10 min, followed by pipetting 100 μL DI water to mix with 5 mg TEMP. The mixture was irradiated with 808 nm NIR light for 10 min.

### 2.8. Antibacterial Test

Gram-positive *S. aureus* (ATCC6538) and gram-negative *E. coli* (ATCC8739) obtained from the Guangdong Microbial Culture Collection Center were employed to assess the antibacterial activity of the various samples. For the Hemin-CaP powder, 160 μL Hemin-CaP suspension (2 mg/mL) was mixed with 640 μL bacterial suspension (10^7^ CFU/mL) in a 24-well plate. Bacterial suspension mixed with 160 μL DI water was set as the control group. Then, the samples were tested with or without 808 nm NIR light (1.6 W/cm^2^) irradiation for 30 min. For the coatings, samples were immersed in 800 μL bacterial suspension in the 24-well plate for 40 min with or without irradiation of 808 nm NIR light. Subsequently, the bacterial suspension was diluted 1000 times with 0.85% NaCl solution and spread onto a beef extract peptone agar medium (0.5% NaCl, 1% peptone, 0.5% beef extract and 2% agar). The number of bacterial colonies was counted after the agar plates were incubated at 37 °C for 18 h.

In order to observe the bacterial morphology using SEM (Apreo S HiVac, Thermo Fisher Scientific, USA), bacteria in the samples were fixed in 2.5 v% glutaraldehyde solution, dehydrated in ethanol solution and dried in tertiary butanol ethanol solution [50].

### 2.9. Cytotoxicity Test

MC3T3-E1 pre-osteoblasts (Wuhan Union Hospital) were utilized to assess the cytotoxicity of various samples via an MTT assay [51]. Moreover, a live/dead staining experiment was applied to discriminate cell morphology [52]. The specific procedures are provided in the supporting information.

### 2.10. Statistical Analysis

The data from the antibacterial and cytotoxicity tests, presented as mean ± standard deviation, were obtained for *n* = 3. *t*-tests were conducted to ascertain the significance level (* *p* < 0.05, ** *p* < 0.01, *** *p* < 0.001). *p* < 0.05 was considered a significant difference.

## 3. Results

### 3.1. Surface Analysis

**Figure 2:**
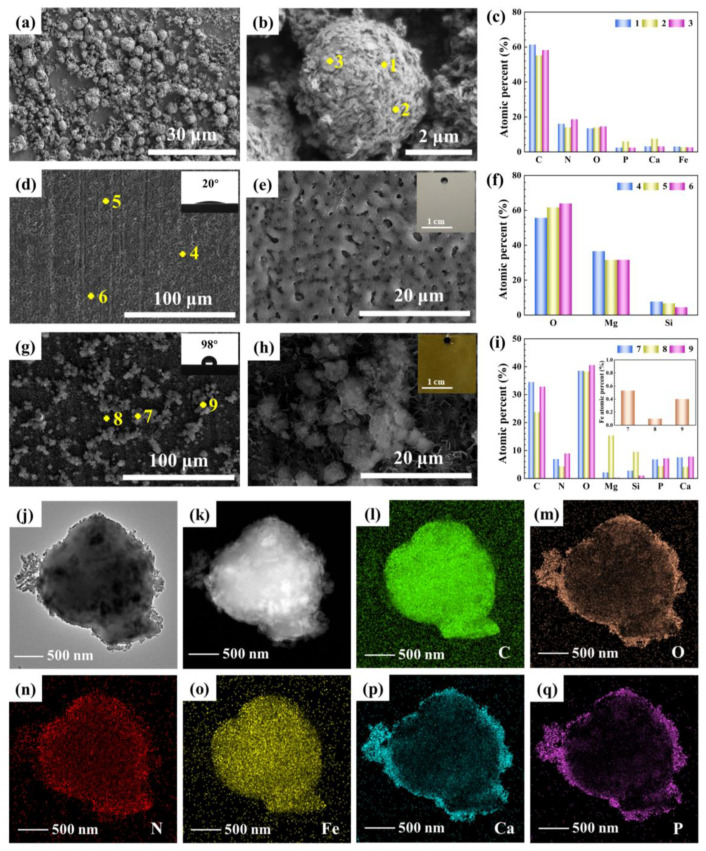
SEM images and corresponding EDS spectra of (**a**–**c**) Hemin-CaP powder, (**d**–**f**) MAO and (**g**–**i**) MAO/PMTMS@(Hemin-CaP) coatings (insets in (**d**) and (**g**) are water contact angles of coatings, insets in e and h are macrographs of coatings and inset in (**i**) is EDS spectrum of Fe element); (**j**) high resolution transmission electron microscopy (HRTEM) and (**k**) high-angle annular dark-field-scanning transmission electron microscopy (HAADF-STEM) images of Hemin-CaP powder; (**l**–**q**) element maps for C, O, N, Fe, Ca and P in Hemin-CaP powder. The element maps were collected from the same area as the HAADF-STEM image in (**k**).

SEM images of Hemin-CaP powder are shown in Figure 2a,b. The powder possessed sphere-like morphology with a diameter of 2–4 μm covered by flake structures. The corresponding EDS data exhibited that the powder contained C, N, O, P, Ca and Fe elements (Figure 2c). The Ca/P ratio was about 1.27, and the N/Fe ratio greater than 4, which may be ascribed to the demetallization of partial porphyrins during the solvothermal process. The MAO coating had typical porous structures (Figure 2d,e). The main elemental composition of the MAO coating was Mg, O and Si (Figure 2f), indicating the existence of MgO and MgSiO_3_. Furthermore, the MAO/PMTMS@(Hemin-CaP) coating appeared as flower-like structures with a cluster of particles distributed on the coating surface (Figure 2g,h). The presence of Fe, N, Ca and P confirmed the loading of Hemin-CaP powder (Figure 2i). The contact angle measurements showed that the MAO coating had a highly hydrophilic surface with a contact angle of 20° (Figure 2d), as the water droplet easily wetted and spread out over the MAO film and quickly penetrated into its porous structures. In comparison, the MAO/PMTMS@(Hemin-CaP) coating exhibited a hydrophobic property with a contact angle of 98° (Figure 2g), indicating that the polymer coating could effectively diminish its contact area with the corrosive aqueous solution, which would be beneficial to improve its corrosion resistance. TEM images further determined that the flake structures of Hemin-CaP powder were calcium-bearing phosphates, and the elements C, N and Fe were mainly situated in the nuclei; that is, hemin was packaged into the microspheres (Figure 2j–q).

**Figure 3:**
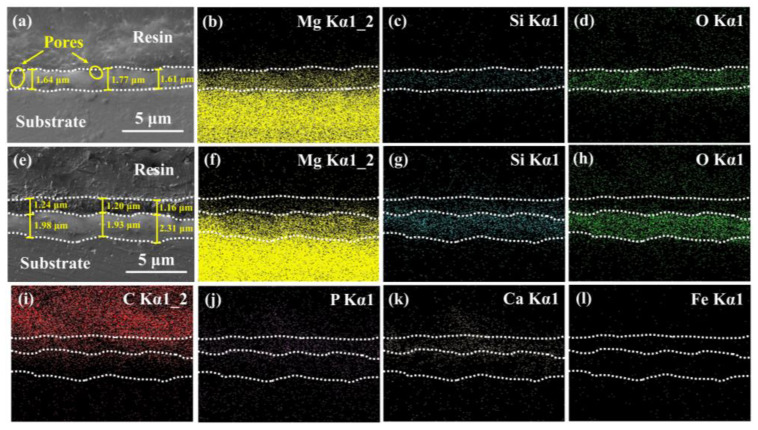
Cross-sectional and corresponding EDS mapping images of (**a**–**d**) MAO and (**e**–**l**) MAO/PMTMS@(Hemin-CaP) coatings.

The cross-sectional morphologies and corresponding EDS mapping images of the MAO and MAO/PMTMS@(Hemin-CaP) coatings are displayed in Figure 3. The thickness of the MAO coating filled with Mg, Si and O elements was 1.67 ± 0.09 μm (Figure 3a–d). Micropores of various dimensions were located in the MAO coating. Nevertheless, the MAO/PMTMS@(Hemin-CaP) coating can be divided into an outer loose layer (1.20 ± 0.04 μm) loaded with Hemin-CaP microspheres and an inner dense layer (2.07 ± 0.21 μm) with MAO coating sealed by PMTMS (Figure 3e), which would be confirmed by the elemental signals of Mg, O, Ca and P. Note that the Si signal in the outer layer also proved the successful preparation of PMTMS (Figure 3g).

**Figure 4:**
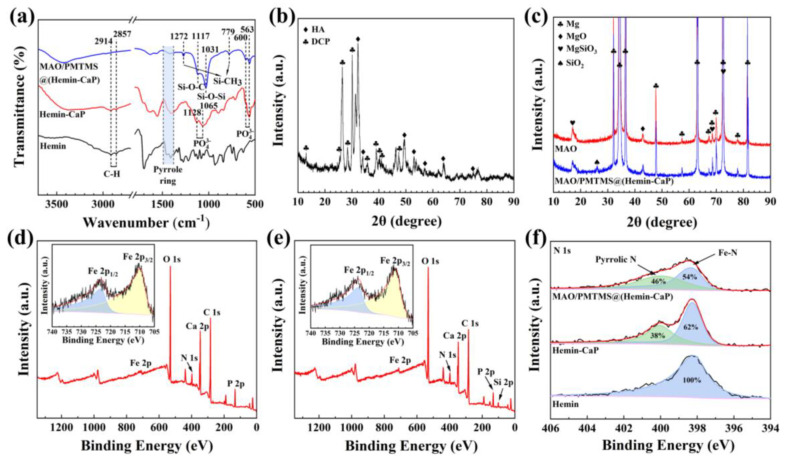
(**a**) FT-IR spectra of pure hemin, Hemin-CaP powder and MAO/PMTMS@(Hemin-CaP) coating; XRD patterns of (**b**) Hemin-CaP powder, (**c**) MAO and MAO/PMTMS@(Hemin-CaP) coatings; XPS overview spectra of (**d**) Hemin-CaP powder and (**e**) MAO/PMTMS@(Hemin-CaP) coating (insets are the XPS spectra of Fe 2p); (**f**) XPS spectra of N 1s of pure hemin, Hemin-CaP powder and MAO/PMTMS@(Hemin-CaP) coating.

The FT-IR spectrum of the samples is depicted in Figure 4a. For pure hemin, the bands of C-H stretching of alkyl groups were found at 2914 and 2857 cm^−1^ [53]. The peaks around 1380–1500 cm^−1^ arose from the skeletal vibrations of the tetrapyrrolyic macrocycle [54]. In addition to the peaks of C-H and tetrapyrrolyic macrocycle associated with hemin, the peaks correlated with PO_4_^3-^ could be observed at 1128, 1065, 600 and 563 cm^−1^ in the Hemin-CaP powder [12,55,56,57]. For the MAO/PMTMS@(Hemin-CaP) coating, the peaks associated with the porphyrin ring and PO_4_^3-^ were exhibited at 1380–1500, 600 and 563 cm^−1^, demonstrating that hemin and CaP were successfully loaded. Moreover, the existence of Si-CH_3_ (1272 and 779 cm^−1^), Si-O-C (1117 cm^−1^) and Si-O-Si (1031 cm^−1^) groups supported the formation of PMTMS [22,24]. The XRD pattern in Figure 4b further proved that the main components of CaP in Hemin-CaP powder were HA and DCP. As displayed in Figure 4c, diffraction peaks of Mg, MgO and MgSiO_3_ were observed, confirming the construction of the MAO coating. The peak at 2θ = 26° observed in the MAO/PMTMS@(Hemin-CaP) coating demonstrated the presence of an amorphous Si-O-Si structure [22]. The C 1s, N 1s, O 1s, P 2p, Ca 2p and Fe 2p signals were observed in the XPS spectrum of Hemin-CaP powder in Figure 4d, which is in agreement with the EDS results in Figure 2c. Furthermore, an extra Si 2p signal was detected in the XPS spectrum of the MAO/PMTMS@(Hemin-CaP) coating (Figure 4e). For ascertaining the changes in the hemin structure of Hemin-CaP powder and composite coating, the XPS spectra of N 1s of pure hemin, Hemin-CaP powder and composite coating are displayed in Figure 4f. For pure hemin, the four chemically equivalent N atoms were coordinated with the central Fe atom, and only a single peak appeared at 398.3 eV in the N 1s spectrum [35,58,59]. However, the N 1s spectrum of Hemin-CaP powder and composite coating had an additional pyrrolic N peak with an area percentage of 38% and 46% at 400.0 eV, respectively, indicating that the demetallization of porphyrins occurred continuously during the preparation of powder and coating [60,61].

### 3.2. Corrosion Characterization

**Figure 5:**
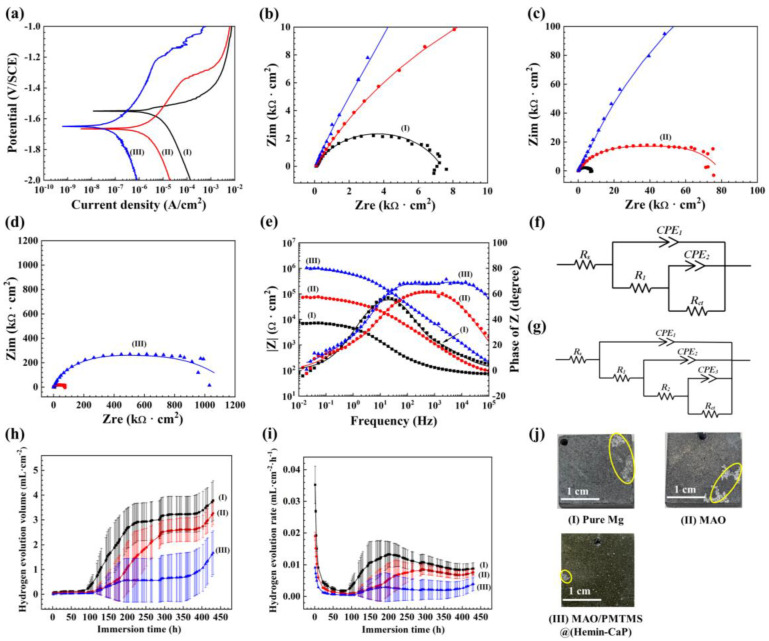
(**a**) Potentiodynamic polarization curves, (**b**–**d**) Nyquist plots, (**e**) Bode plot of |Z| vs. frequency and Bode plot of phase angle vs. frequency of (Ⅰ) pure Mg, (Ⅱ) MAO coating and (Ⅲ) MAO/PMTMS@(Hemin-CaP) coating in Hank’s solution; the corresponding EC models of (**f**) pure Mg, MAO and (**g**) MAO/PMTMS@(Hemin-CaP) coatings; (**h**) HEV curves, (**i**) HER curves and (**j**) photographs of (Ⅰ) pure Mg, (Ⅱ) MAO and (Ⅲ) MAO/PMTMS@(Hemin-CaP) coatings with an immersion of 430 h in Hank’s solution.

The potentiodynamic polarization curves and corresponding electrochemical parameters of (Ⅰ) pure Mg, (Ⅱ) MAO and (Ⅲ) MAO/PMTMS@(Hemin-CaP) coatings are depicted in Figure 5a and Table S1, respectively. The *i*_corr_ of MAO/PMTMS@(Hemin-CaP) coating was decreased to 4.41 × 10^−8^ A·cm^−2^ in comparison with pure Mg (3.12 × 10^−6^ A·cm^−2^) and the MAO coating (9.67 × 10^−7^ A·cm^−2^), demonstrating that the composite coating possessed the best corrosion resistance. Additionally, the composite coating exhibited continuous passivation behavior in the anodic region, which is related to the fracture and reconnection of the Si-O-Si bond of silane [24]. The *R*_p_ of the composite coating (5.68 × 10^5^ Ω·cm^2^) was one order of magnitude higher than that of the MAO coating (2.60 × 10^4^ Ω·cm^2^), indicating that the composite coating can protect the pure Mg better than the MAO coating.

The Nyquist plots of the samples are shown in Figure 5b–d. The diameter of the capacitive loop of the MAO/PMTMS@(Hemin-CaP) coating was observably larger than that of the MAO coating and pure Mg, confirming that the composite coating provided a more effective physical barrier [62], which could be attributed to the sealing effect of PMTMS and the thicker composite coating (Figure 3e). The Bode plot in Figure 5e revealed that the low-frequency impedance modulus |Z| of the MAO/PMTMS@(Hemin-CaP) coating was higher than that of pure Mg and the MAO coating, which is in accordance with the analysis of Nyquist plots. As for the phase angle plot, the time constant of pure Mg in the middle-frequency region was associated with the formation of Mg(OH)_2_ corrosion product [63]. Since a ceramic MgO layer was formed on pure Mg, the time constant of the MAO coating in the middle-frequency region became wider [22,63,64]. For the composite coating, two time constants were observed, which can be associated with its bilayer structure.

The EC models in Figure 5f,g were used to further explain the corrosion behavior of the samples, and the corresponding fitting results are listed in Table S2. In the EC models, *R*_s_ and *R*_ct_ are the solution resistance and the charge transfer resistance, respectively. *R*_1_ and *R*_2_ represent the film resistance. *CPE* represents the constant potential element and *n* is the degree to which the element deviates from pure capacitance. *CPE* is the same as capacitance when *n* = 1, Warburg impedance when *n* = 0.5 and resistance when *n* = 0 [65]. For pure Mg in Hank’s solution, two *RQ* circuits can be found in Figure 5f, indicating that a corrosion product film formed on the substrate. The same EC model also appeared in the MAO coating because of the porous oxide layer. The *R*_1_ of pure Mg (1.62 × 10^2^ Ω·cm^2^) was much lower than that of the MAO coating (4.64 × 10^4^ Ω·cm^2^), suggesting that the corrosion product film of pure Mg was relatively loose and thin. As displayed in Figure 5g, the EC model of the MAO/PMTMS@(Hemin-CaP) coating was composed of three *RQ* circuits related to substrate, inner layer and outer layer, respectively. Since the outer layer of the composite coating was loose while the inner layer was dense, the outer layer resistance *R*_1_ (6.65 × 10^4^ Ω·cm^2^) was one order of magnitude lower than the inner layer resistance *R*_2_ (4.75 × 10^5^ Ω·cm^2^), agreeing with the cross-sectional morphology in Figure 3e. The composite coating had the maximum *R*_ct_ value (6.37 × 10^5^ Ω·cm^2^), implying its positive effect on corrosion protection.

The hydrogen evolution volume (HEV) and hydrogen evolution rate (HER) curves of the various samples immersed in Hank’s solution for 430 h are shown in Figure 5h,i. For the pure Mg, a corrosion product film rapidly formed on substrate in the initial 80 h causing a reduced HER. During 80–200 h, the corrosion product film disintegrated and its HEV and HER increased gradually. The degradation and formation of corrosion products reached a dynamic equilibrium during 200–380 h, which led to a relatively stable HEV. Similarly, there was also a period of corrosion product formation in the initial 110 h for the MAO coating. Since the corrosive medium penetrated into the porous structures of MAO, HEV and HER increased after immersion for 110 h. With the continuous formation of corrosion products, HEV tended to be stable at about 300–380 h. As for the MAO/PMTMS@(Hemin-CaP) coating, both the overall HEV and HER of the composite coating were lower than that of the MAO coating since the micropores of the MAO coating were completely sealed. The slight increase in HEV after 110 h of immersion might be owing to the involvement of Fe^3+^ ions released from porphyrin by galvanic corrosion. Nevertheless, the HEV became flat after immersion for 190 h as the corrosion products were ceaselessly accumulated. Note that the HEV of pure Mg, MAO and composite coatings rose after 380 h, indicating that their corrosion product films were penetrated by the corrosive medium again. Due to the galvanic corrosion of the composite coating, its slope of HER was larger than that of pure Mg and the MAO coating. The photographs of the various samples immersed for 430 h are displayed in Figure 5j. There were large corrosion defects on the surface of pure Mg and the MAO coating, while the composite coating was not greatly damaged except for a small number of corrosion pits. Both electrochemical and hydrogen evolution tests showed that the composite coating had excellent corrosion resistance.

**Figure 6:**
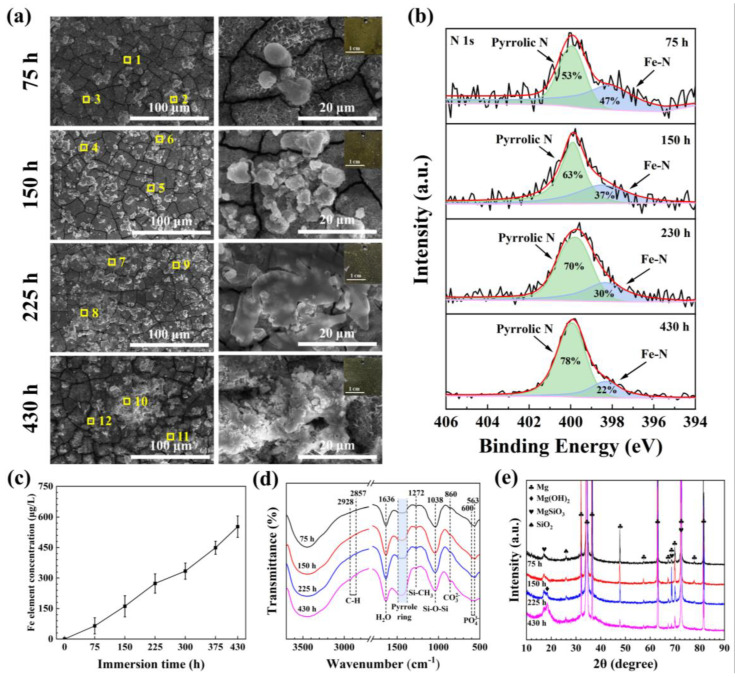
(**a**) SEM images of MAO/PMTMS@(Hemin-CaP) coating with immersions of 75, 150, 225 and 430 h (insets are macrographs of coatings); (**b**) XPS spectra of N 1s of MAO/PMTMS@(Hemin-CaP) coating at various immersion times; (**c**) atomic absorption spectroscopy of Fe element released from the MAO/PMTMS@(Hemin-CaP) coating at various immersion times; (**d**) FT-IR spectra and (**e**) XRD patterns of the sample with different immersion times.

The morphologies and corresponding EDS spectra of the MAO/PMTMS@(Hemin-CaP) coating with immersions of 75, 150, 225 and 430 h are displayed in Figure 6a and Supplementary Figure S1. With a 75 h immersion, parts of the microspheres were degraded and corrosion cracks were observed. The flake structures covered on the microspheres disappeared, which would facilitate the release of Fe^3+^ ions. After an immersion of 150 h, a small number of corrosion products was formed. After immersing for 225 h, the corrosion products were increased and piled up together. Until 430 h, the cracks on the coating surface were enlarged, further accelerating the corrosion process. It can be seen from the FT-IR spectrum in Figure 6d that the peak of CO_3_^2−^ progressively appeared with the increase in immersion time due to the dissolution of CO_2_, while the peaks of silane and porphyrin ring were still existing. A new Mg(OH)_2_ peak appeared after 225 h of immersion in the XRD pattern (Figure 6e), revealing that the main corrosion product of the composite coating after 430 h immersion was Mg(OH)_2_, and the major structures of the composite coating were not changed significantly. In order to ascertain the variation in the hemin structure in the MAO/PMTMS@(Hemin-CaP) coating during immersion, XPS spectra of N 1s with immersions of 75, 150, 225 and 430 h are shown in Figure 6b. The peak area ratio of pyrrolic N to Fe-N progressively increased with the increase in immersion time, revealing that more and more Fe atoms were removed from the porphyrin macrocycles during the immersion process. The relation between Fe content in Hank’s solution and immersion time is displayed in Figure 6c. It was slowly released from the composite coating without burst release, which is consistent with the XPS results. Owing to the essential roles of Fe in the human body, the continuous release of Fe might have a positive effect on cell growth, which will be confirmed in the following cytotoxicity test.

### 3.3. Photothermal and Photodynamic Effects

**Figure 7:**
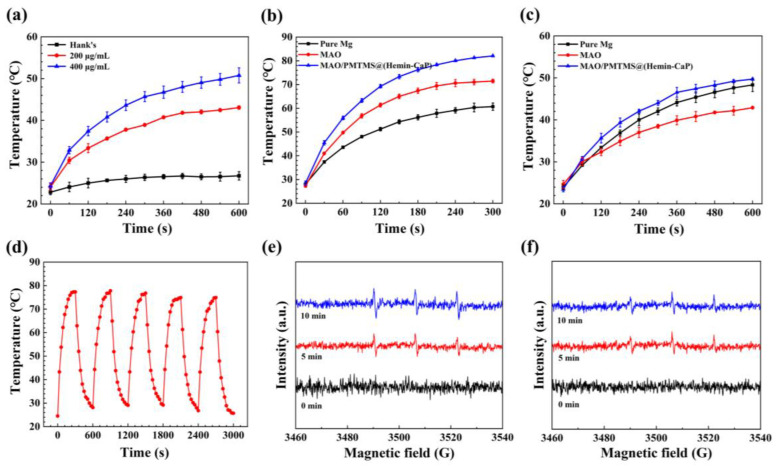
(**a**) Temperature change curves of Hemin-CaP suspensions under 808 nm NIR irradiation at different concentrations; temperature change curves of pure Mg, MAO and MAO/PMTMS@(Hemin-CaP) coatings (**b**) in air and (**c**) in Hank’s solution under 808 nm NIR irradiation; (**d**) photothermal on-off effect of MAO/PMTMS@(Hemin-CaP) coating in air; ESR spectra of (**e**) Hemin-CaP powder and (**f**) MAO/PMTMS@(Hemin-CaP) coating after 808 nm NIR irradiation for 10 min.

The photothermal properties of Hemin-CaP with different dispersion concentrations were investigated under NIR irradiation, as displayed in Figure 7a. It was obvious that the final temperature after 10 min irradiation increased with increasing concentration of Hemin-CaP microspheres. The final temperature reached 51 °C when the concentration was 400 μg/mL. The photothermal heating curves of pure Mg, MAO and MAO/PMTMS@(Hemin-CaP) coatings are depicted in Figure 7b,c. In an air environment, the surface temperature of the MAO/PMTMS@(Hemin-CaP) coating reached 82 °C after irradiation for 5 min. In Hank’s solution, the surface temperature of the composite coating was 50 °C, approximately 7 °C higher than that of the MAO sample after irradiation for 10 min. Note that the final temperature of pure Mg was even higher than that of the MAO coating. This phenomenon may be attributed to the two reasons below. Firstly, the thermal conductivity of pure Mg is higher than that of MAO [32], which benefited the transfer of heat from the surface of the substrate to the solution. Secondly, the increased temperature of the solution further promoted the corrosion of pure Mg and produced heat [36,66]. As shown in Figure 7d, the temperature of the MAO/PMTMS@(Hemin-CaP) coating exhibited a stable on-off behavior. These results revealed a favorable photothermal effect of the MAO/PMTMS@(Hemin-CaP) coating. After calculation, the *η* of the MAO/PMTMS@(Hemin-CaP) coating was about 20%, where *τ*_s_ and *A_λ_* were 414.68 and 0.255, respectively (Figure S2). In order to explore the photodynamic effect of samples, ESR was utilized to detect the ^1^O_2_ produced by Hemin-CaP powder and the MAO/PMTMS@(Hemin-CaP) coating, as shown in Figure 7e,f. For the Hemin-CaP powder, no ^1^O_2_ signal was detected without irradiation. However, the trapped ^1^O_2_ signal increased gradually as the irradiation time increased from 0 to 10 min. In addition, the ^1^O_2_ signal was also detected in the MAO/PMTMS@(Hemin-CaP) coating owing to the loading of the powder, which proved its photodynamic effect.

### 3.4. Antibacterial Ability

**Figure 8:**
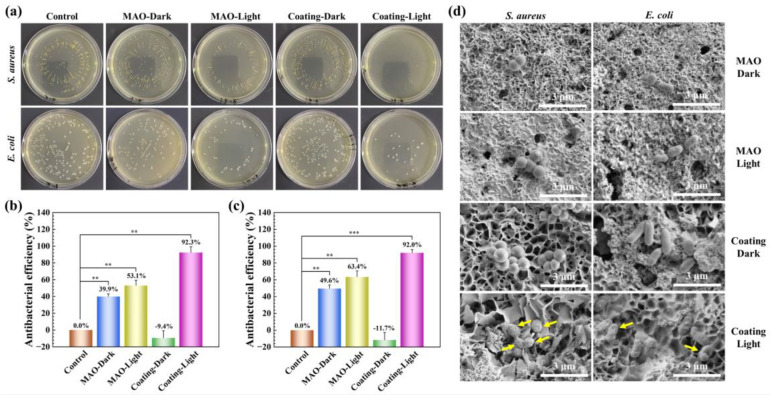
In vitro antibacterial activity of MAO and MAO/PMTMS@(Hemin-CaP) coatings against *S. aureus* and *E. coli* in the dark or under 808 nm NIR irradiation (1.6 W/cm^2^) for 40 min: (**a**) representative images of bacterial colonies; corresponding antibacterial efficiency against (**b**) *S. aureus* and (**c**) *E. coli* and (**d**) SEM images of bacterial morphologies. (** *p* < 0.01, *** *p* < 0.001).

The antibacterial properties of the Hemin-CaP powder, MAO and MAO/PMTMS@(Hemin-CaP) coatings against *S. aureus* and *E. coli* in the dark or under NIR irradiation are presented in Figure S3 and Figure 8. Considering that the temperature change of pure Mg in solution under NIR irradiation may be related to its accelerated corrosion, the release of excessive Mg^2+^ and OH^-^ ions would greatly inhibit the bacterial activity [6,31]. Therefore, a control group and an MAO sample were used for comparison with the MAO/PMTMS@(Hemin-CaP) coating. For the Hemin-CaP powder, the number of bacterial colonies under dark conditions was higher than that of the control group, demonstrating its excellent biocompatibility. Fortunately, the antibacterial efficiency of the powder against *S. aureus* and *E. coli* under NIR irradiation reached as high as 99.9% and 98.3%, respectively. The antibacterial ability of the MAO/PMTMS@(Hemin-CaP) coating had the same trend as the powder. Under NIR irradiation, its antibacterial efficiency against *S. aureus* and *E. coli* was 92.3% and 92.0%, respectively. In fact, the MAO coating also had a certain antibacterial ability, which can be attributed to its increased pH value [36]. The increased temperature of the MAO coating after exposure to NIR light also led to an improved antibacterial efficiency.

Furthermore, the bacterial morphologies on the MAO and MAO/PMTMS@(Hemin-CaP) coatings were observed with SEM, as displayed in Figure 8d. Under dark conditions, the composite coating surface had a considerable number of bacteria with intact morphology. However, under NIR light irradiation, significant deformation of the bacteria could be observed, suggesting that membrane damage occurred in the bacteria, which led to the leakage of cellular contents and bacterial death [67]. Han et al. [68] found that when the bacteria were mixed with the porphyrin-containing metal-organic frameworks (MOFs) and exposed to light in an ice-bath, damage to the membrane was weaker. However, when the bacteria were mixed with MOFs and treated with light at room temperature, the combination of ^1^O_2_ and heat was much more effective in causing membrane damage. As described in Section 3.3, the MAO/PMTMS@(Hemin-CaP) coating was capable of generating heat and ^1^O_2_ under NIR irradiation. Although ^1^O_2_ could amplify intracellular oxidative stress leading to structural damage of nucleic acids and proteins [69], in the present study, the bacteria were killed mainly by heat supplemented by the synergistic effect of ^1^O_2_ due to the weak ^1^O_2_ signal of the coating (Figure 7f).

### 3.5. Cytotoxicity Test

**Figure 9:**
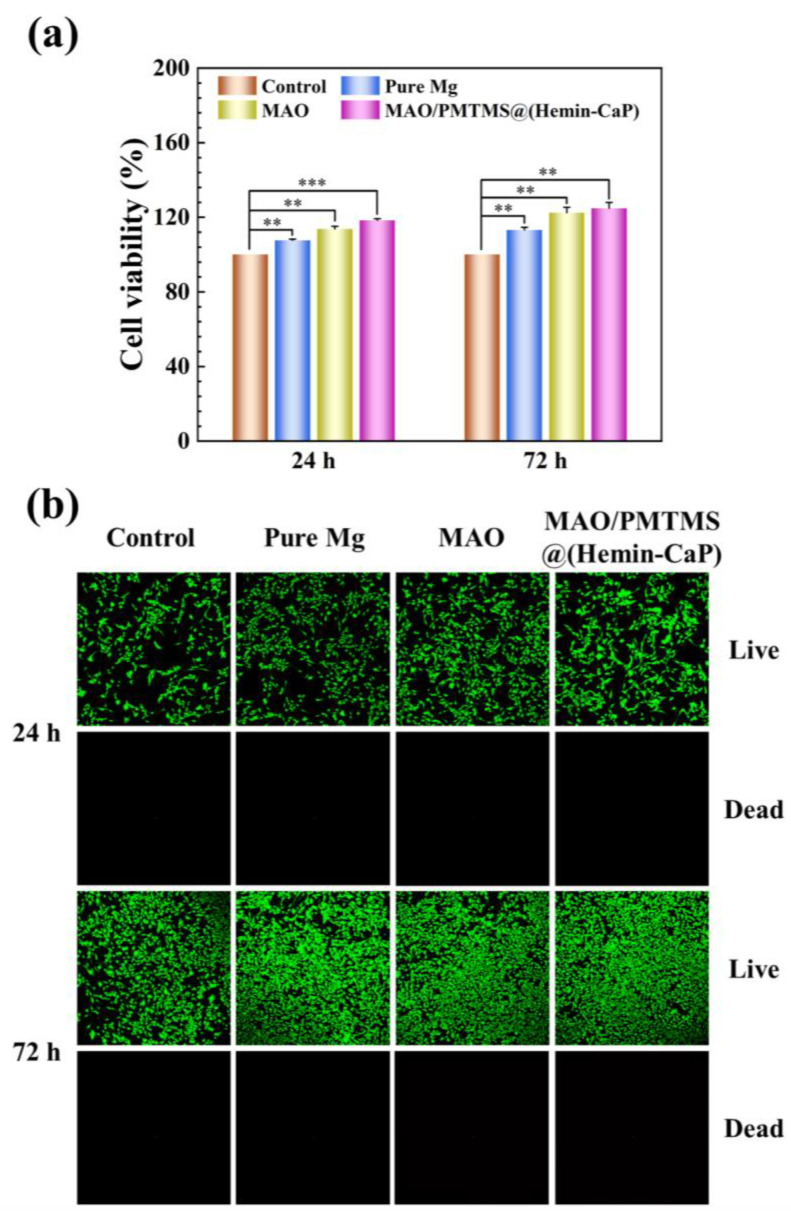
(**a**) Cell viability and (**b**) CLSM images of MC3T3-E1 after 24 and 72 h of culture in the 72 h extracts at 20% concentration prepared with pure Mg, MAO and MAO/PMTMS@(Hemin-CaP) coatings. (** *p* < 0.01, *** *p* < 0.001).

Figure 9a exhibits the cell viability of MC3T3-E1 after 24 and 72 h of culture in the 72 h extracts prepared with various samples. After 24 h incubation, the cell viability of pure Mg and MAO coating was 108% and 114%, respectively, suggesting the osteogenic effect of Mg^2+^ ions [4]. As for the MAO/PMTMS@(Hemin-CaP) coating, the cell viability significantly increased to 118% and further reached up to 125% after culturing for 72 h, indicating a positive effect of the coating in promoting bone growth. The remarkable rise in cell viability may be related to the release of Fe^3+^ ions from the coating due to the vital role of Fe element in cell proliferation and differentiation [70]. Additionally, CLSM images of MC3T3-E1 of various groups are displayed in Figure 9b. The cells for all groups exhibited a broadly healthy fusiform-like shape and displayed a high degree of spreading regarding morphology.

## 4. Discussion

### 4.1. Comparison of Coatings on Mg-Based Bone Implants

**Figure 10:**
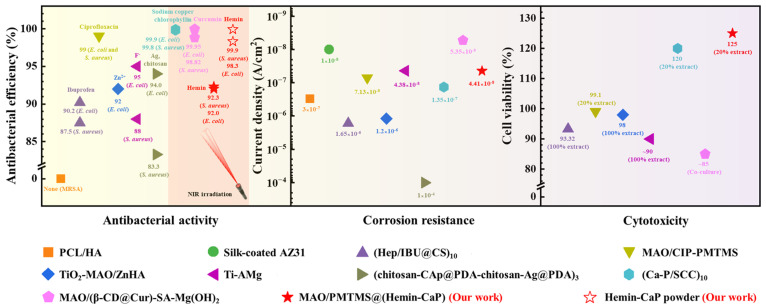
Summary of corrosion resistance, antibacterial activity and cytotoxicity of Mg-based bone implants [23,35,36,72,73,74,75,76,77].

IAI after orthopedic surgery is an arduous challenge. Therefore, it is particularly important to focus on improving the corrosion resistance, biocompatibility and antibacterial activity of Mg-based implants. The comparison of the above three properties of Mg-based implant coatings is summarized in Figure 10. Although conventional polymer coatings can improve the corrosion resistance and biocompatibility of Mg alloys, they possess almost no antibacterial ability. One of the ways to enhance antibacterial ability is through loading drugs or adding antibacterial metallic elements. However, the release of drugs and antibacterial elements, especially the initial burst release, may be detrimental to the growth of cells. In addition, the presence of metallic elements in the coating will unavoidably lead to galvanic corrosion and accelerate the failure of the coatings. In particular in our prior research [35], a sodium copper chlorophyllin induced calcium-bearing phosphate coating was prepared on the Mg alloy AZ31 and obtained a good antibacterial activity due to the combination of PDT, PTT and Cu^2+^ ions. Usually, the larger the electrode potential difference between the two metals, the greater the severity of galvanic corrosion [71]. The standard electrode potential of Mg (*E*(Mg^2+^/Mg) = −2.372 V) is much lower than that of Cu (*E*(Cu^2+^/Cu) = 0.3419 V); that is, the Mg substrate with a lower electrode potential becomes more active and corrodes preferentially. Thus, the burst release of Cu^2+^ ions from the coating during a 24 h immersion led to the initial failure of the coating. With a 75 h immersion, the surface of the coating had obvious corrosion cracks. However, in this work, the electrode potential difference between Fe (*E*(Fe^3+^/Fe) = −0.037 V) in hemin and the Mg substrate is smaller and the induced flaky CaP and adsorbed PMTMS limit the release rate of Fe^3+^ ions from porphyrins, resulting in a reduced galvanic corrosion. In addition, a proper release rate of Fe^3+^ ions also plays an important role in promoting pre-osteoblast growth. Therefore, the composite coating prepared in this research exhibits good antibacterial ability, long-term corrosion resistance and excellent biocompatibility. However, owing to the insufficient loading of microspheres, the *η* and antibacterial efficiency of the coating need to be further improved by replacing synthetic polymers with better a encapsulation ability as carriers. The aim is to increase the loading of hemin while maintaining the corrosion resistance of the coating.

### 4.2. Coating Formation and Degradation Mechanisms

The formation of the MAO/PMTMS@(Hemin-CaP) coating can be summarized as the hydrolysis condensation of silane and the adsorption of Hemin-CaP powder. The first step is that MTMS generates hydroxyl structures through the following hydrolysis reaction:

(2) $${\left({\mathrm{CH}}_{3}O\right)}_{3}{\text{-Si-CH}}_{3}{+3H}_{2}O\;\to \;{\left(\mathrm{HO}\right)}_{3}{\text{-Si-CH}}_{3}{+3\mathrm{CH}}_{3}\mathrm{OH}$$

After that, the hydroxylated MTMS combines with the hydroxyl groups on the surface of the NaOH-pretreated MAO coating by dehydration condensation. Moreover, the (HO)_3_-Si-CH_3_ structures are capable of self-condensation with each other to form a Si-O-Si chain. Since the microspheres have positively charged calcium sites on their surface, they are able to physically adsorb with negatively charged hydroxyl groups.

According to the results of the hydrogen evolution test, the degradation process of the MAO/PMTMS@(Hemin-CaP) coating can be approximately classified into three stages:

(1) After the sample is immersed in Hank’s solution, the microspheres adsorbed on the surface of the MAO/PMTMS@(Hemin-CaP) coating will be continuously degraded. As the flake structures covering the microspheres disappear, the exposed hemin nuclei and the demetallation of hemin will facilitate the occurrence of galvanic corrosion. Since PMTMS acts as a physical barrier, the hydrogen evolution of the sample is not obvious in the early period of immersion.
(2) The corrosion cracks and the permeability of PMTMS become channels for the corrosive medium to penetrate the inner MAO, resulting in a slight release of hydrogen. As the corrosion products continually accumulate in the cracks of the coating, the contact between the solution and substrate is suppressed for a short period of time. The HEV is relatively flat during this period.
(3) With the immersion time extension, the corrosion products become loose and the corrosive medium is once again in contact with the substrate. The galvanic corrosion between Fe^3+^ ions and the Mg matrix leads to a rapid increase in HEV.

### 4.3. Antibacterial Activity and Biocompatibility of the Coating

**Figure 11:**
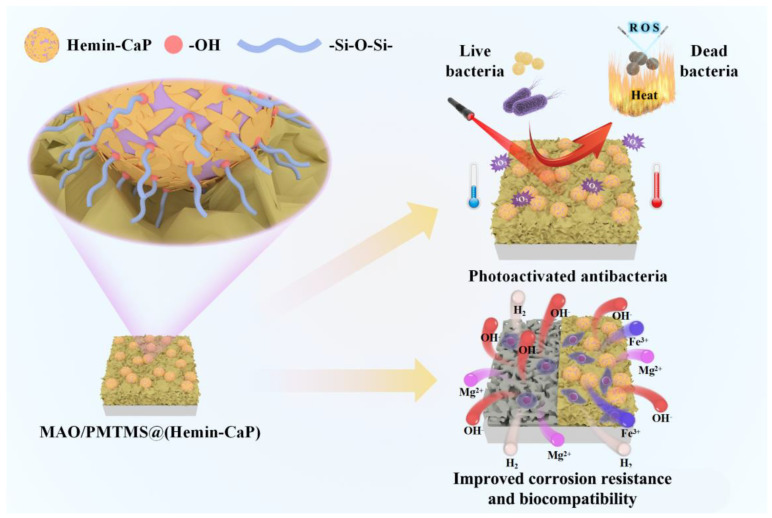
Illustration of formation mechanism of MAO/PMTMS@(Hemin-CaP) coating and its photoactivated antibacterial activity, improved corrosion resistance and biocompatibility.

Hemin-CaP powder and MAO/PMTMS@(Hemin-CaP) coating exhibit photothermal and photodynamic effects, which are mainly attributed to the porphyrin macrocycles of hemin [37]. During co-incubation with bacteria, porphyrin macrocycles were excited using NIR to generate heat and ROS, causing membrane damage in bacteria and leading to their death (Figure 8d). In addition, the composite coating also exhibited the ability to promote the growth of MC3T3-E1 pre-osteoblasts, which is related to the proper release of Mg^2+^ and Fe^3+^ ions during the degradation of the coating (Figure 6c). The mechanisms of the antibacterial property and biocompatibility of the coating are illustrated in Figure 11.

## 5. Conclusions

A MAO/PMTMS@Hemin-CaP coating was successfully prepared on pure Mg via MAO processing and dipping treatments.

(1) The inner nuclei of Hemin-CaP microspheres are hemin and the outer flake structures are DCP and HA. After 808 nm NIR irradiation, the antibacterial efficiency of the Hemin-CaP powder against *S. aureus* and *E. coli* reaches as high as 99.9% and 98.3%, respectively, which is attributed to its good photothermal and photodynamic properties.
(2) The MAO/PMTMS@(Hemin-CaP) coating appears as flower-like structures and a cluster of microspheres distributed on the coating surface via physical adsorption. The *i*_corr_ of the MAO/PMTMS@(Hemin-CaP) coating (4.41 × 10^−8^ A·cm^−2^) is two orders of magnitude lower than that of pure Mg (3.12 × 10^−6^ A·cm^−2^), indicating the composite coating can provide excellent corrosion protection. Flaky CaP and PMTMS act as physical barriers, restrict the release of Fe^3+^ ions and prevent the penetration of the corrosion medium, alleviating galvanic corrosion of the Mg substrate.
(3) By virtue of the photothermal and photodynamic performance of the Hemin loaded by microspheres, the antibacterial efficiency of the MAO/PMTMS@(Hemin-CaP) coating against *S. aureus* and *E. coli* under 808 nm NIR irradiation is 92.3% and 92.0%, respectively.
(4) The MC3T3-E1 pre-osteoblasts cultured in the 72 h extracts prepared with the MAO/PMTMS@(Hemin-CaP) coating exhibit a broadly healthy fusiform-like shape and display a high degree of spreading regarding morphology. The cell viability is 125%, indicating a positive effect of the coating in promoting bone growth, which is promising for application as bone implants.

## Data Availability

Not applicable.

## Supplementary Materials

The following supporting information can be downloaded at: https://www.mdpi.com/article/10.3390/jfb14010015/s1, Figure S1. EDS spectra of MAO/PMTMS@(Hemin-CaP) coating with an immersion of 75, 150, 225 and 430 h (insets are EDS spectra of Fe element); Figure S2. (**a**) Heating/cooling curve of MAO/PMTMS@(Hemin-CaP) coating in Hank’s solution under 808 nm irradiation (1.6 W/cm^2^) and corresponding (**b**) linear fitting of the cooling period vs. −lnθ; (**c**) UV-vis absorption spectra of the various samples; Figure S3. In vitro antibacterial activity of Hemin-CaP against (**a**) *S. aureus* and (**b**) *E. coli* in the dark or under 808 nm irradiation (1.6 W/cm^2^) for 30 min. The corresponding bacterial colony counts of (**c**) *S. aureus* and (**d**) *E. coli*; Table S1. Electrochemical parameters of the potentiodynamic polarization curves; Table S2. Electrochemical data obtained via equivalent circuit fitting of EIS curves.
