# Supplementary material for: In Vitro Degradation and Photoactivated Antibacterial Activity of a Hemin-CaP Microsphere-Loaded Coating on Pure Magnesium
Source: J Funct Biomater. 2022 Dec 26;14(1):15. doi: 10.3390/jfb14010015 (PMC9861195; doi:10.3390/jfb14010015)
Supplement: Supplementary file 1 [file jfb-14-00015-s001.zip › jfb-2106435-supplementary.pdf]

Supporting Information

In Vitro Degradation and Photoactivated Antibacterial Activity of a Hemin-CaP Microsphere-Loaded Coating on Pure Magnesium

Lixin Long [1], Yang Song [1], Xiaoyi Tian [1], Lanyue Cui [1,*], Chengbao Liu [1], Shuoqi Li [1], Yu Wang [2,*] and Rongchang Zeng [1,3]

[1] College of Materials Science and Engineering, Shandong University of Science and Technology, Qingdao 266590, China

[2] College of Pharmaceutical Sciences, Soochow University, Suzhou 215123, China

[3] School of Materials Science and Engineering, Zhengzhou University, Zhengzhou 450002, China

[*] Corresponding authors.

E-mail addresses: cuilanyue2010@126.com (L.C.); yuwang@suda.edu.cn (Y.W.)

EXPERIMENTAL SECTION

Calculation of the photothermal conversion efficiency

The photothermal conversion efficiency (η) of MAO/PMTMS@(Hemin-CaP) coating was calculated according to previous literature [1]. The total energy balance for this system can be expressed as following:

$$\sum_i m_i C_{p,\,i}\frac{\mathrm{d}T}{\mathrm{d}t} = Q_{\mathrm{Sample}} + Q_{\mathrm{s}} - Q_{\mathrm{loss}} \tag{1}$$

where m and C_p are the mass and heat capacity, respectively. The suffix "i" of m and C_p refers to water or pure Mg. T is the solution temperature.

Q_{Sample} is the photothermal energy input by MAO/PMTMS@(Hemin-CaP) coating per second:

$$Q_{\mathrm{Sample}} = I\left(1 - 10^{-A_\lambda}\right)\eta \tag{2}$$

where I is the laser power, A_λ is the absorbance of MAO/PMTMS@(Hemin-CaP) coating at the wavelength of 808 nm.

Q_{loss} is thermal energy lost to the surroundings:

$$Q_{\mathrm{loss}} = hA\Delta T \tag{3}$$

where h is the heat transfer coefficient, A is the surface area of the container, and ΔT is defined as T - T_{surr} (T and T_{surr} are the solution temperature and ambient temperature of the surrounding, respectively).

Q_{s} is the heat associated with the light absorbed by solvent per second. In the situation of irradiating pure water, the heat input is equal to the heat output at the maximum steady-statue temperature:

$$Q_{\mathrm{s}} = Q_{\mathrm{loss}} = hA\Delta T_{\mathrm{max,\,H_2O}} \tag{4}$$

where $\Delta T_{\mathrm{max,\,H_2O}}$ is the temperature change of water at the maximum steady-state temperature.

For the photothermal experiment of the MAO/PMTMS@(Hemin-CaP) coating, the heat inputs are the heat generated by the sample (Q_{Sample}) and the heat generated by water (Q_{S}), which is equal to the heat output at the maximum steady-statue temperature:

$$Q_{\mathrm{Sample}} + Q_{\mathrm{s}} = Q_{\mathrm{loss}} = hA\Delta T_{\mathrm{max,\,mix}} \tag{5}$$

where $\Delta T_{\mathrm{max,\,mix}}$ is the temperature change of the MAO/PMTMS@(Hemin-CaP) coating at the maximum steady-state temperature. According to the equation (2), (4) and (5), the photothermal conversion efficiency can be expressed as following:

$$\eta = \frac{hA(\Delta T_{\text{max, mix}} - \Delta T_{\text{max, H}_2\text{O}})}{I(1 - 10^{-A_\lambda})} \tag{6}$$

In order to get the hA, a dimensionless driving force θ and a sample system time constant τ_s are introduced:

$$\theta = \frac{\Delta T}{\Delta T_{\text{max}}} \tag{7}$$

$$\tau_s = \frac{\sum_i m_i C_{p,\,i}}{hA} \tag{8}$$

Substituting equation (7) and (8) into equation (1):

$$\frac{\mathrm{d}\theta}{\mathrm{d}t} = \frac{1}{\tau_s}\left[\frac{Q_{\text{Sample}} + Q_s}{hA\Delta T_{\text{max}}} - \theta\right] \tag{9}$$

When the laser was shut off, $Q_{\text{Sample}} + Q_s = 0$, equation (9) changed to:

$$t = -\tau_s \ln\theta \tag{10}$$

where τ_s can be calculated by linear fitting of the cooling period versus $-\ln\theta$.

Cytotoxicity test

Before the experiment, all samples were sterilized by UV irradiation for 30 min. Then they were immersed in dulbecco's modified eagle medium (DMEM) supplemented with 10% fetal bovine serum (FBS) in a humidified atmosphere with 5% CO_2 at 37 °C. After immersing for 72 h, the supernatant fluid was filtered using a 0.22 μm filter. A 96-well plate was utilized to culture the seeded cells at a density of 5×10^3/well to allow attachment. Afterwards, the cell culture fluid was changed to 100 μL of DMEM supplemented with 20% extracts and 80% fresh cell medium. With a culture of 24 and 72 h, the cell culture medium was removed. Successively, each well was washed three times with phosphate buffered saline (PBS), added 100 μL of medium containing 0.5 mg/mL 3-(4, 5-dimethyl-2-thiazolyl)-2, 5-diphenyltetrazolium bromide (MTT) and incubated in a 37 °C incubator under 5% CO_2 for 4 h. Subsequently, 100 μL dimethyl sulfoxide (DMSO) was added to dissolve the formazan crystals produced during the incubation period. After shaking for 10 min, the absorbance at a wavelength of 570 nm was detected.

Furthermore, live/dead staining experiment was employed to discern cell morphology. In brief, a confocal petri dish was used to incubate the pre-osteoblasts with a density of 2×10^4/well. After 6 h, the culture medium was changed to 1000 μL of DMEM supplemented with 20% extracts and 80% fresh cell medium for experimental groups and regular cell culture media for control group. Afterwards, cells were cultured in a humidified atmosphere with 5% CO_2 at 37 °C for 24 h. After

washing confocal petri dish with PBS to remove the excess of FBS, 500 μL staining solution with the composition of calcein-AM and propidium iodide (PI) was added to co-culture with cell for 30 min in a 37 °C incubator under 5% CO_2. Subsequently, cell morphology was observed via confocal laser scanning microscope (CLSM, LSM710, Carl Zeiss, German).

RESULTS

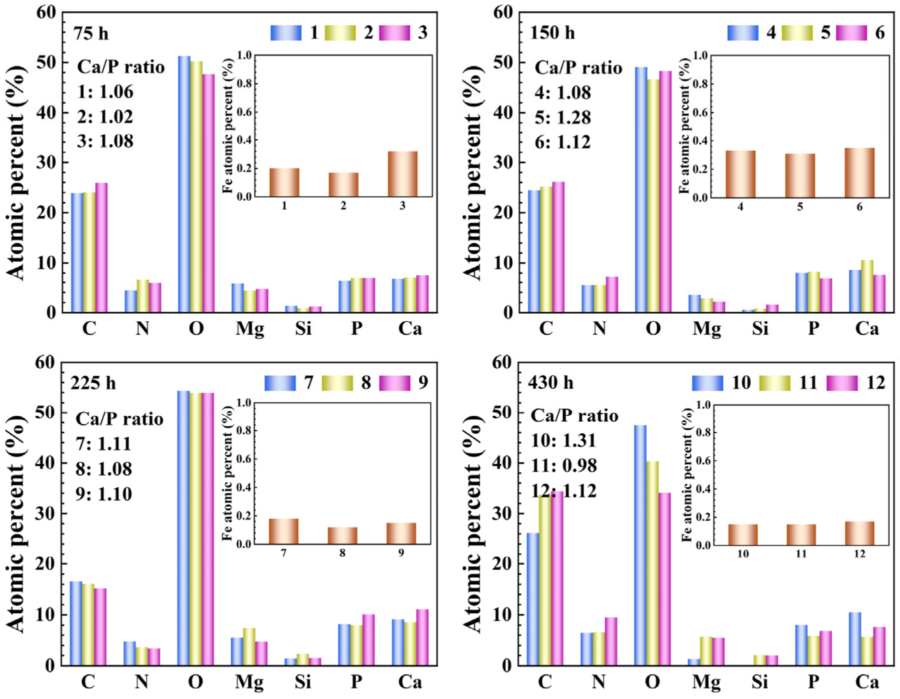

Figure S1. EDS spectra of MAO/PMTMS@(Hemin-CaP) coating with an immersion of 75, 150, 225 and 430 h (insets are EDS spectra of Fe element).

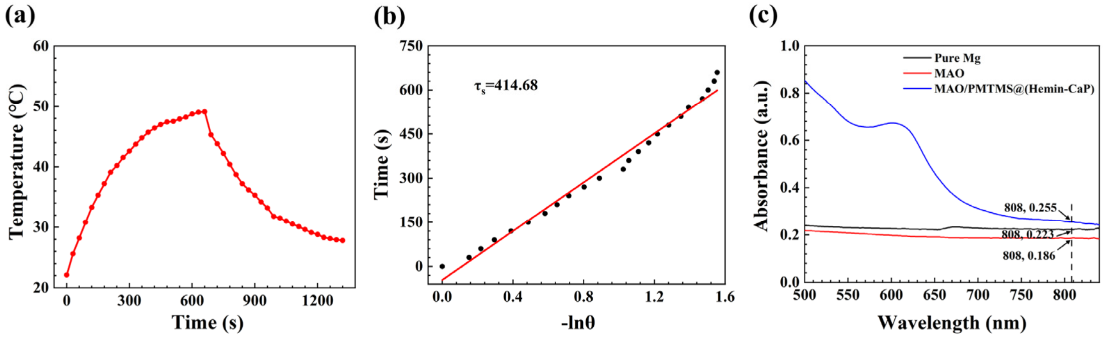

Figure S2. (**a**) Heating/cooling curve of MAO/PMTMS@(Hemin-CaP) coating in Hank's solution under 808 nm irradiation (1.6 W/cm^2) and corresponding (**b**) linear fitting of the cooling period vs. -lnθ; (**c**) UV-vis absorption spectra of the various samples.

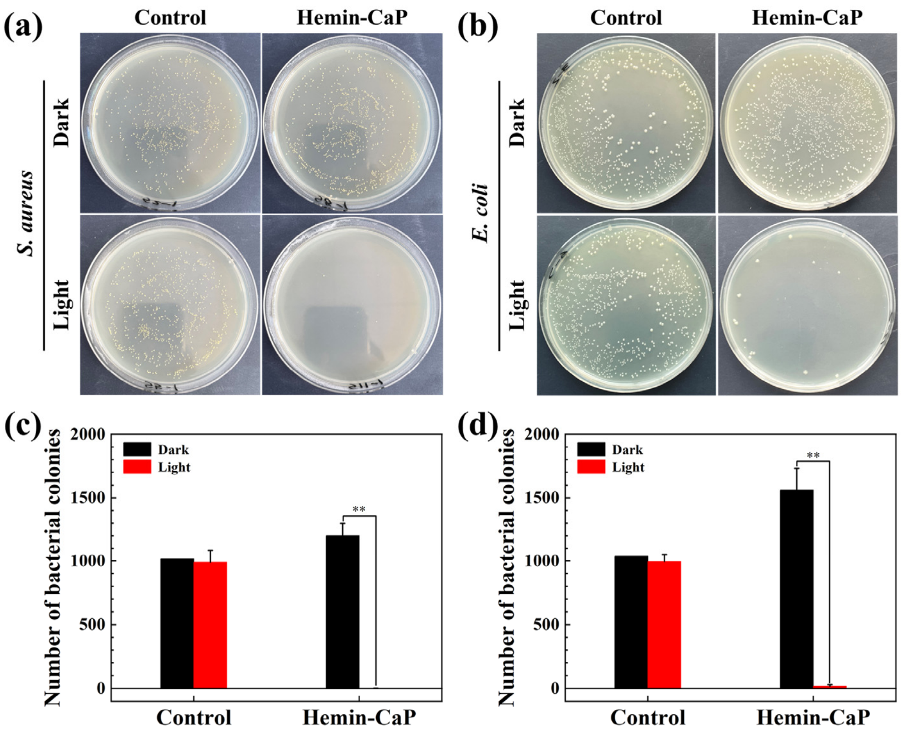

Figure S3. *In vitro* antibacterial activity of Hemin-CaP against (**a**) *S. aureus* and (**b**) *E. coli* in dark or under 808 nm irradiation (1.6 W/cm^2) for 30 min. The corresponding bacterial colonies counts of (**c**) *S. aureus* and (**d**) *E. coli*.

Table S1. Electrochemical parameters of the potentiodynamic polarization curves.

**Samples**	E_{corr} (V)	i_{corr} (A·cm^{-2})	β_a (mV·dec^{-1})	-β_c (mV·dec^{-1})	R_p (Ω·cm^2)
Pure Mg	-1.55	3.12×10^{-6}	64.66	129.50	6.00×10^{3}
MAO	-1.67	9.67×10^{-7}	96.59	145.16	2.60×10^{4}
MAO/PMTMS@ (Hemin-CaP)	-1.65	4.41×10^{-8}	95.25	146.29	5.68×10^{5}

Table S2. Electrochemical data obtained via equivalent circuit fitting of EIS curves.

**Samples**	**Pure Mg**	**MAO**	**MAO/PMTMS@ (Hemin-CaP)**
R_s (Ω·cm^2)	7.22×10^{1}	6.98×10^{1}	6.00×10^{1}
CPE_1 (Ω^{-1}·s^n·cm^{-2})	2.53×10^{-5}	1.67×10^{-6}	1.20×10^{-7}
n_1	0.67	0.72	0.80
R_1 (Ω·cm^2)	1.62×10^{2}	4.64×10^{4}	6.65×10^{4}
CPE_2 (Ω^{-1}·s^n·cm^{-2})	8.59×10^{-6}	1.85×10^{-5}	1.52×10^{-8}
n_2	0.85	0.69	0.91
R_2 (Ω·cm^2)	-	-	4.75×10^{5}
CPE_3 (Ω^{-1}·s^n·cm^{-2})	-	-	1.37×10^{-6}
n_3	-	-	0.58
R_{ct} (Ω·cm^2)	7.10×10^{3}	3.28×10^{4}	6.37×10^{5}
Chi	1.88×10^{-3}	1.52×10^{-3}	3.30×10^{-3}

REFERENCES

1. Tian, Q.W.; Jiang, F.R.; Zou, R.J.; Liu, Q.; Chen, Z.G.; Zhu, M.F.; Yang, S.P.; Wang, J.L.; Wang, J.H.; Hu, J.Q. Hydrophilic Cu_9S_5 nanocrystals: A photothermal agent with a 25.7% heat conversion efficiency for photothermal ablation of cancer cells *in vivo*. *ACS Nano* **2011**, *5*, 9761-9771, doi:10.1021/nn203293t.
